# VAPEX: an interactive web server for the deep exploration of natural virus and phage genomes

**DOI:** 10.1093/bioinformatics/btad528

**Published:** 2023-08-25

**Authors:** Benjamin Hepp, Florence Lorieux, Augustin Degaugue, Jacques Oberto

**Affiliations:** Institute for Integrative Biology of the Cell (I2BC), CEA, CNRS, Université Paris‐Saclay, 91198 Gif‐sur‐Yvette cedex, France; Institute for Integrative Biology of the Cell (I2BC), CEA, CNRS, Université Paris‐Saclay, 91198 Gif‐sur‐Yvette cedex, France; Institute for Integrative Biology of the Cell (I2BC), CEA, CNRS, Université Paris‐Saclay, 91198 Gif‐sur‐Yvette cedex, France; Institute for Integrative Biology of the Cell (I2BC), CEA, CNRS, Université Paris‐Saclay, 91198 Gif‐sur‐Yvette cedex, France

## Abstract

**Motivation:**

Studying the genetic makeup of viruses and phages through genome analysis is crucial for comprehending their function in causing diseases, progressing medicine, tracing their evolutionary history, monitoring the environment, and creating innovative biotechnologies. However, accessing the necessary data can be challenging due to a lack of dedicated comparative genomic tools and viral and phage databases, which are often outdated. Moreover, many wet bench experimentalists may not have the computational proficiency required to manipulate large amounts of genomic data.

**Results:**

We have developed VAPEX (Virus And Phage EXplorer), a web server which is supported by a database and features a user-friendly web interface. This tool enables users to easily perform various genomic analysis queries on all natural viruses and phages that have been fully sequenced and are listed in the NCBI compendium. VAPEX therefore excels in producing visual depictions of fully resolved synteny maps, which is one of its key strengths. VAPEX has the ability to exhibit a vast array of orthologous gene classes simultaneously through the use of symbolic representation. Additionally, VAPEX can fully analyze user-submitted viral and phage genomes, including those that have not yet been annotated.

**Availability and implementation:**

VAPEX can be accessed from all current web browsers such as Chrome, Firefox, Edge, Safari, and Opera. VAPEX is freely accessible at https://archaea.i2bc.paris-saclay.fr/vapex/.

## 1 Introduction

Viruses are small infectious agents whose replication occurs obligatorily inside the cells of living organisms and relies on cellular metabolism. They are composed of genetic material (either DNA or RNA) surrounded by a protein capsid. Enveloped viruses harbor an extra lipid bilayer membrane surrounding the capsid. Viruses infect virtually any eukaryal or archaeal cell. Bacteria are not immune from viral infection and their viruses are called phages, short for bacteriophages.

Viruses cause a number of human diseases such as influenza, AIDS, Ebola, and SARS-CoV2, and pose a constant threat to public health. The occurrence of viral epidemics threatens also all varieties of cultivated plants to sustain human population and its domesticated animals (Jones 2020, Verhagen *et al.* 2021).The study of viruses is of crucial importance to understand how they cause disease and to develop strategies to understand and prevent the mechanisms of viral transmission. The monitoring of viruses present in many environments is therefore essential to measure the spread of these viral infections and controlling outbreaks. Outside of medicine, other harmful aspects include the constant threat of bacteriophage contamination in industrial processes involving bacterial fermentation, such as food production and pharmaceutical manufacturing (Karczewska *et al.* 2023). On the positive side, biotechnological approaches generated cures for viral diseases using Louis Pasteur’s weakened forms of viruses (Smith 2012) and recombinant or mRNA vaccines (Janowski and Andrzejewska 2022). Phage therapy or the use of bacteriophages to cure bacterial infections in humans is gaining renewed interest as a potential alternative to antibiotics in the face of antibiotic resistance (Lin *et al.* 2017). Finally, studying viruses is important to understand their evolution and the interactions with their hosts. Viruses are among the most ancient and abundant organisms on Earth, and they have shaped the evolution of all forms of life (Koonin 2010). All aforementioned virus and phage studies clearly benefit from resources dedicated to their classification and to the analysis of their genomes. A number of public repositories have been developed toward the descriptive comparison, taxonomy and classification of viruses such as ICTV (Lefkowitz *et al.* 2018), NCBI viral genomes resource (Brister *et al.* 2015), ViralZone (Hulo *et al.* 2011), and BV-BRC (Olson *et al.* 2023). Other public access resources permit the exploration of evolutionary relationships among prokaryotic viruses such as PHROG (Terzian *et al.* 2021) which provides a classification of proteins in orthologous groups or Phagonaute (Delattre *et al.* 2016) which displays the genetic context of selected genes across prokaryotic viruses within the internal database.

To our knowledge, there is currently a lack of public web services that provide comprehensive genomic comparisons of bacteriophages, eukaryotic viruses, and archaeal viruses especially when user-submitted genomes are involved. As a solution, we introduce VAPEX—a user-friendly and interactive web server aimed at facilitating in-depth examination of natural virus and phage genomes including user-submitted sequences. By utilizing the well-established symbolic representation developed for the WASPS web server (Badel *et al.* 2020), VAPEX can create comprehensive and fully resolved synteny maps for both bioinformaticians and wet lab experimentalists.

## 2 Methods

The VAPEX web server is composed of three interconnected modules: the VAPEX database, the VAPEX web application and the VAPEX database Updater program.

**VAPEX database:** All VAPEX viral and phage data are contained in a relational database stored locally on a dedicated server. The database adopts a hierarchical linked list structure of virus/phage objects, each carrying several fields for accession numbers, definitions, genome size, and a gene protein list object. Moreover, the database incorporates extra fields to accommodate future expansions. The gene protein list object carries all gene entries relative to a specific genome with the following fields: accession, definition, orientation, coordinates, orthologous cluster id, and centroid accession. The accession field of each gene protein is appended to the accession field of the originating genome for tracking purposes. All virus/phage and gene fields are extracted from the NCBI GenBank files with the exception of the orthologous gene cluster and centroid fields which are calculated locally at database creation or regeneration. Genes predicted by VAPEX are annotated using centroid definitions. On the server, the VAPEX core database consists of an XML file that is well-suited for storing linked lists. To improve performance, the XML file is deserialized into memory objects in the server’s RAM during the first access. Virus/phage DNA sequences, the translation of coding and centroid genes reside in three separate binary files. Accession numbers link the core database to DNA and protein sequences. At this writing, the VAPEX database carries 15.288 virus/phage genomes corresponding to 631.296 protein sequences ranked in 143.677 orthologous clusters.

**VAPEX updater:** A separate single executable stored on the server carries out at regular intervals all tasks required for a fully automated database update. NCBI GenBank refseq files are retrieved from ftp://ftp.ncbi.nih.gov/refseq/release/viral/ using the FTP protocol and parsed to extract the data to populate the fields of the core database mentioned above. DNA and protein sequences are extracted as well and merged into two FASTA files. The orthologous relationships between proteins sequences are calculated using MMseqs2 (Mirdita *et al.* 2019) which generates a list of orthologous clusters and a FASTA file compiling the centroid protein sequences from each cluster. The three FASTA files corresponding to genomic DNA, protein genes and protein centroids are then converted into BLAST-compatible binary format using MakeBlastDB (Altschul *et al.* 1990) and Diamond-compatible format with Makedb (Buchfink *et al.* 2015). The database update pipeline is depicted in Supplementary Material.

**VAPEX web server:** The interface of the VAPEX web server allows the submission of a variety of queries to its database. Context-sensitive hypertext links leading to the help file are provided for all functions and options. VAPEX salient features are listed below:

1. User-submitted annotated virus/phage genomes are compared to the database in order to generate fully resolved synteny maps in nearly real time. The protein sequences are extracted from the annotation and compared with the binary centroid database using BLAST (Altschul *et al.* 1990). Matching proteins are assigned the corresponding VAPEX cluster number. Collinear synteny maps comprising the query genome and matching VAPEX genomes are then drawn to scale with individual open reading frames colored according to cluster membership. To allow for the simultaneous display of protein genes from a large number of clusters, the coloring is obtained with a combination of symbols and hues. The *E*-values of predicted matching protein sequences are indicated on the synteny map.
2. Unannotated virus/phage sequences can be submitted as well. In this case, VAPEX predicts protein coding capacity in the six possible reading frames and these potential open reading are treated as described above. The query genome is then displayed in the synteny map using six collinear tracks.
3. Smooth user navigation in the synteny maps by the means of zoom, pan and scroll is accomplished with typical mouse/touchpad gestures. Gene identification tooltips are obtained by hovering. Map navigation is executed entirely using HTML 5.0 canvas properties of modern browsers and does not require data transfer from the server.
4. Text queries are performed on the accession and definition fields of all virus/phage genomes and protein genes. Matching results are linked to synteny queries.
5. User-submitted DNA or protein sequences are matched against the VAPEX database using a choice of BLAST (Altschul *et al.* 1990) and Diamond (Buchfink *et al.* 2015) algorithms. Synteny links are provided for all matches.
6. All results generated by VAPEX can be exported as text, scalable vector graphics (SVG) or bitmap (PNG) formats for further analysis.

## 3 Results

**Figure 1.**
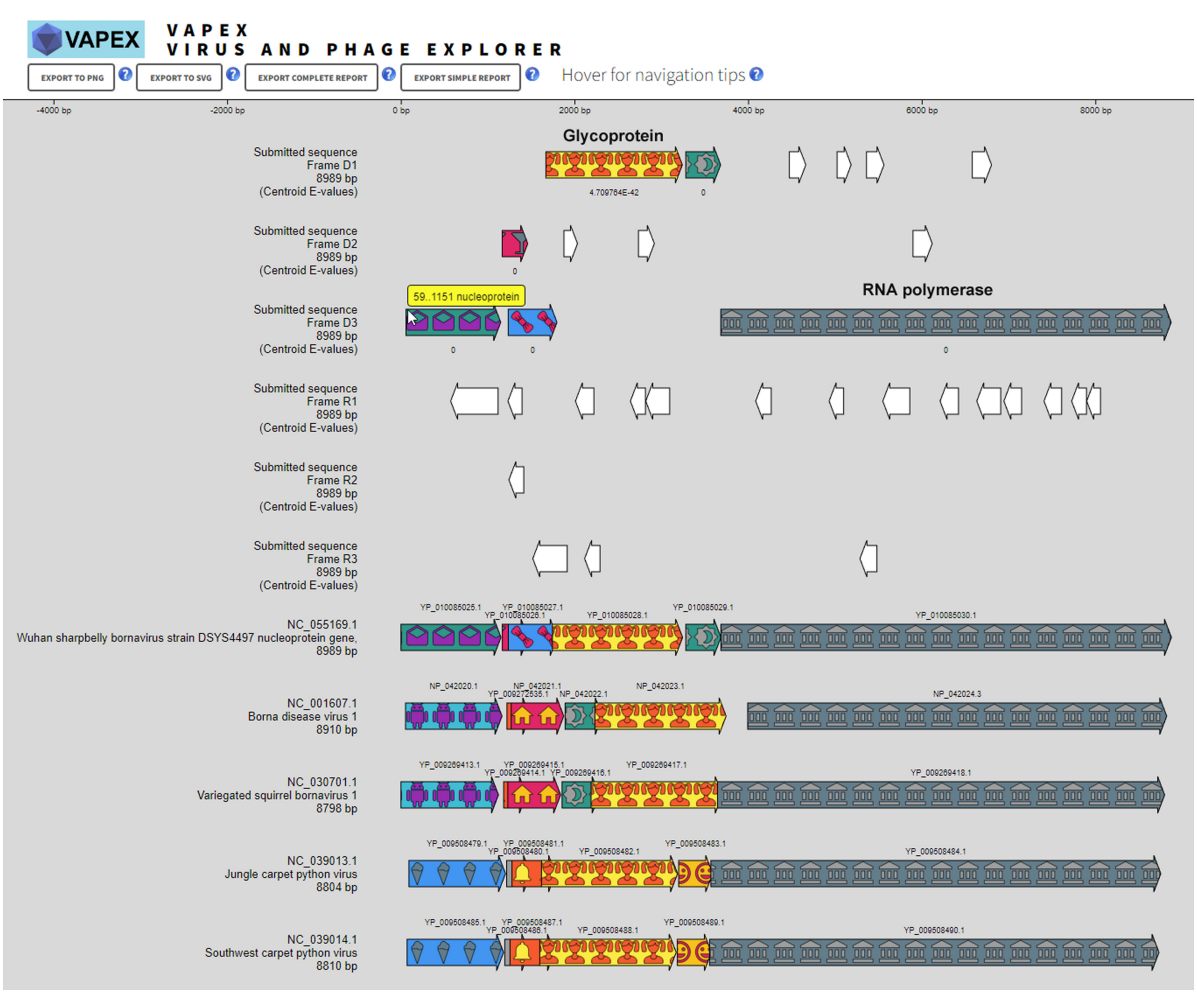
Synteny analysis of the Wuhan sharpbelly bornavirus. The first six tracks refer to the open reading frames predicted by VAPEX in the submitted unannotated FASTA DNA sequence from GenBank entry NC_055169.1. The following tracks correspond to related viruses detected in the VAPEX database using BLAST with the “extended cluster hits” option. Low predicted *E*-values are due to the presence of NC_055169.1 in the database. Consistent gene symbol and coloring identify the orthologous clusters of the corresponding proteins. Syntenies involving partial genomes sequences present in the database were omitted for clarity.

Recent events have highlighted the significance of tracing the origins of RNA viruses, such as SARS-CoV-2 that has caused a global pandemic or Ebola that has led to highly fatal epidemics. The onset of the Ebola virus disease outbreak in West Africa between 2013 and 2016 signaled the inception of widespread real-time molecular epidemiology (Holmes *et al.* 2016). Molecular phylogenetic techniques that analyze genetic sequences directly, such as maximum likelihood and Bayesian methods, are generally considered the most effective in identifying the origins and evolution of sporadically emerging pathogenic viruses. In this study, we examined whether VAPEX-generated gene order conservation or synteny analyses could serve as a reliable and effective technique for tracking the evolutionary history of related viral genomes. VAPEX predictive orthology capabilities were assessed by comparing RNA Filoviruses which include the pathogenic Marburg and Ebola viruses. Due to sampling biases, these RNA viruses were though initially to be restricted to mammalian and avian hosts. Recently, a comprehensive transcriptomic survey of a diverse range of vertebrates revealed the presence of distantly related *Filoviridae* not only in mammals and birds but also in fish and amphibians (Shi *et al.* 2018). Due to its nucleoprotein belonging to a distinct orthologous cluster, the Wuhan sharpbelly bornavirus, a virus that infects freshwater fish, has been classified as a phylogenetic outgroup in *Filoviridae* (Shi *et al.* 2018). We were therefore compelled to analyze the genomic sequence of this bornavirus with VAPEX. When querying the corresponding unannotated FASTA nucleotide sequence from NC_055169.1, multiple open reading frames were detected and identified easily by conducting a synteny analysis with other viruses stored in the VAPEX database (Fig. 1). As expected, VAPEX readily confirmed that the nucleoprotein of the bornavirus belongs to a distinct orthologous cluster in comparison to other related *Filoviridae*. Surprisingly, the RNA-dependent RNA polymerase and glycoprotein encoded by the Wuhan sharpbelly bornavirus belong to the same orthologous groups as the corresponding proteins in related viruses, suggesting a considerably lower diversity in *Filoviridae* than anticipated. These findings suggested that VAPEX's sensitivity is comparable to that of molecular phylogenetics while offering a broader and more contrastive analysis of the evolution of related viruses.

## Supplementary Material

btad528_Supplementary_Data
