# Supplementary figures and images for: VAPEX: an interactive web server for the deep exploration of natural virus and phage genomes
Source: Bioinformatics. 2023 Aug 25;39(8):btad528. doi: 10.1093/bioinformatics/btad528 (PMC10471898; doi:10.1093/bioinformatics/btad528)

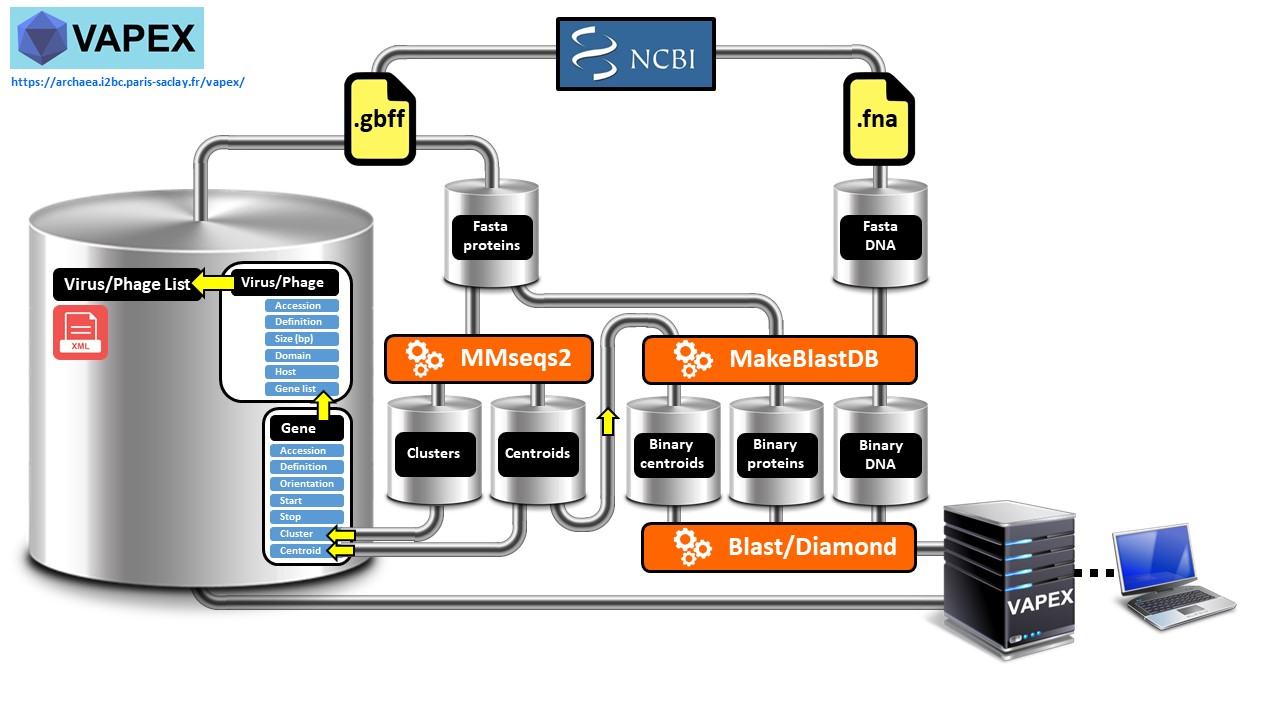

Supplement: btad528_Supplementary_Data [file btad528_supplementary_data.jpeg]
